# The Effects of Hyperbaric Oxygen Therapy on Pelvic Radiation Induced Gastrointestinal Complications (Rectal Bleeding, Diarrhea, and Pain): A Meta-Analysis

**DOI:** 10.3389/fonc.2020.00390

**Published:** 2020-04-09

**Authors:** Jun-hua Yuan, Li-min Song, Yuan Liu, Man-wen Li, Qian Lin, Rui Wang, Cai-shun Zhang, Jing Dong

**Affiliations:** ^1^Department of Special Medicine, School of Basic Medicine, Qingdao University, Qingdao, China; ^2^Department of Physiology, School of Basic Medicine, Qingdao University, Qingdao, China

**Keywords:** pelvic radiotherapy, hyperbaric oxygen, rectal bleeding, diarrhea, pain

## Abstract

**Background:** Radiotherapy is a routine treatment for pelvic cancer patients. While it had been proven effective, gastrointestinal side effects remain a concern, impairing the quality of life. A few studies focused on the effects of hyperbaric oxygen (HBO) treatment to alleviate radiation-induced gastrointestinal complications. This meta-analysis aimed to critically review and summarize existing literature, assessing the effectiveness of HBO therapy for the treatment of radiation-induced gastrointestinal side effects.

**Methods:** Medical literature search was performed with PubMed, Cochrane Library, and EMBASE up to March 14, 2019. Literatures about HBO treatment upon patients undergoing pelvic cancer (endometrial, cervix, rectum, or prostate cancers) radiotherapy were collected, and the effects of HBO treatment on radiotherapy-induced gastrointestinal complications were evaluated. A random-effects model was used to calculate the pooled effect size. Subgroup analyses were performed to search for sources of heterogeneity. Publication bias was detected with Funnel plots and Egger's test.

**Results:** Three different radiotherapy-related gastrointestinal complications, including rectal bleeding, diarrhea, and pain, were analyzed after screening. It was revealed that the improvement rates were considerable in rectal bleeding (0.81, 95% CI: 0.74–0.89) and diarrhea (0.75, 95% CI: 0.61–0.90) and slightly in pain (0.58, 95% CI: 0.38–0.79). Subgroup analysis revealed factors that significantly influenced the heterogeneity of rectal bleeding, diarrhea, and pain (evaluation criteria, follow-up time, and scoring system, respectively). No significant publication bias was detected.

**Conclusion:** HBO treatment might have the potential to alleviate radiotherapy-related gastrointestinal complications, including rectal bleeding, diarrhea, and pain, but more data are needed for further conclusions. Other symptoms were not further analyzed, as the number of studies was insufficient. More large-scale and prospective studies are needed for better evaluation of HBO's therapeutic values.

## Introduction

In 2018, 18.1 million people worldwide were diagnosed with cancer. Among the various cancer types, pelvic cancers posed an increasingly significant health burden worldwide, since the most prevalent cancers in male and female both include pelvic cancers (prostate cancer and cervical cancer, respectively) (1). Fortunately, earlier diagnosis and advanced treatments resulted in a significantly increased number of people living with pelvic cancers since the past decades (2).

Pelvic irradiation is a key component of curative treatment of pelvic malignancies, including gynecological, rectal/anal, and urological cancers. While pelvic irradiation had been proven effective in pelvic cancer management, adverse effects were frequently observed and reported as well (3). In fact, pelvic radiation disease (PRD) was formally defined as a disease in 2010, described as non-cancerous tissue injury secondary to radiotherapy with transient or longer-term problems, ranging from mild to severe, which attracted global attention (4). Morris and Haboubi (5) reported DNA damage as the major identified mechanism of PRD, rapid manifestation of PRD in rapid turnover tissues such as the bowel epithelium, and slower manifestation in slower turnover tissues such as the vascular endothelium and connective tissues. The overall pathological effect is a progressive endarteritis and necrosis, ultimately leading to hypoxia and a characteristic tissue fibrosis (6). While epithelial necrosis had been identified as the major contributor to acute radiation reactions and damage of vascular and stromal cells, Fuccio et al. (7) associated chronic injuries with damage of vascular and stromal cells. In clinical practice, PRD is typically classified by affected organ systems, including urinary, reproductive, and gastrointestinal related injuries. Among them, gastrointestinal complications may develop well after the radiotherapy, even after decades. Hence, the improvement of life expectancy will increase the risk of developing this type of complication, which deserves more attention (8). Pelvic radiation-induced gastrointestinal complications are characterized with acute and chronic symptoms: acute symptoms typically include rectal bleeding, diarrhea, abdominal pain, nausea, bloating, and urgency (9), while chronic symptoms include rectal bleeding, abdominal pain, fecal incontinence, urgency, and flatulence. The chronic symptoms may develop following acute symptoms, or arise independently post-radiation therapy (10). It had been observed that PRD-related symptoms were generally underestimated and poorly managed, in spite of their remarkable impairment on quality of life (3).

The current interventions to alleviate acute and chronic adverse gastrointestinal effects following pelvic radiotherapy include pharmacological interventions (mucosal protectants, anti-inflammatory agents, statins, and angiotensin-converting enzyme inhibitors) and non-pharmacological interventions such as probiotics, nutritional interventions, and hyperbaric oxygen (HBO) therapy (11). Among these options, HBO therapy is the only physical treatment that promotes both tissue healing and angiogenesis through improving oxygen and blood supply as well as anti-inflammatory effects (12–14). The utilization of HBO therapy as an effective method to treat radiation-induced tissue damage could be traced back to the early 1970s (15). Recently, it had been utilized as a recognized treatment option for PRD, and systematic reviews concluded that it did not promote cancer growth or recurrence (16, 17). During the HBO treatments, patients are placed in a compression chamber with elevated oxygen levels at increased barometric pressure, allowing oxygen delivery at a greatly increased pressure to the tissues, mobilizing stem cells, promoting tissue healing, and angiogenesis (13, 14).

In spite of the relatively common application of HBO treatment in PRD management, analysis of the real clinical effects remains controversial and scarce. In a 2018 Cochrane review (11), only one article about HBO treatment was introduced. Recently, a randomized, double-blind study reported no benefit from HBO for patients with radiation-induced chronic gastrointestinal symptoms (18), which aroused considerable debate (19–25). Since there is a lack of large-scale randomized control trials (RCTs) to guide the application of HBO treatment in PRD management, this review aims to synthesize the existing data, analyze results of previous reports, and propose further inquiry of HBO's application in radiation-induced gastrointestinal complications.

## Methods

### Search Strategy

This work was based on the systematic reviews and meta-analyses guidelines in 2015 (26). The search strategy was developed for the following databases: Cochrane Library, PubMed, and EMBASE on March 14, 2019. The search used broadly defined medical subject headings (MeSH) for the following terms: “Hyperbaric Oxygenation,” “Radiotherapy,” “Radiation Injuries,” “Gastrointestinal Hemorrhage,” “Constipation,” “Colic,” “Diarrhea,” “Fecal incontinence,” “Flatulence,” “Lactose intolerance,” “Nausea,” “Abdominal Pain,” “Vomiting,” “Weight loss,” “Hemorrhoids,” “Defecation,” and “Weight gain.” Free terms corresponding to the MeSH in PubMed were also searched. The terms of the pelvic radiation-induced gastrointestinal side effects were identified according to a list of gastrointestinal symptoms after pelvic radiotherapy from a review (3). No language nor date restrictions were applied to any of the searches. In addition, the reference lists were also checked during the full text review to identify any supplementary sources and ensure that all relevant studies had been identified.

Inclusion criteria were as follows:

1. Articles were published in English.
2. Original studies were conducted in humans.
3. The patient groups had received pelvic radiotherapy for pelvic cancers.
4. The trial had assessed the effects of HBO on gastrointestinal complications.

Exclusion criteria were as follows:

1. Reviews, meta-analysis, case reports, posts, and comments.
2. Primary malignancy of non-pelvic cavity sites.

### Data Extraction and Quality Assessment

**Figure 1:**
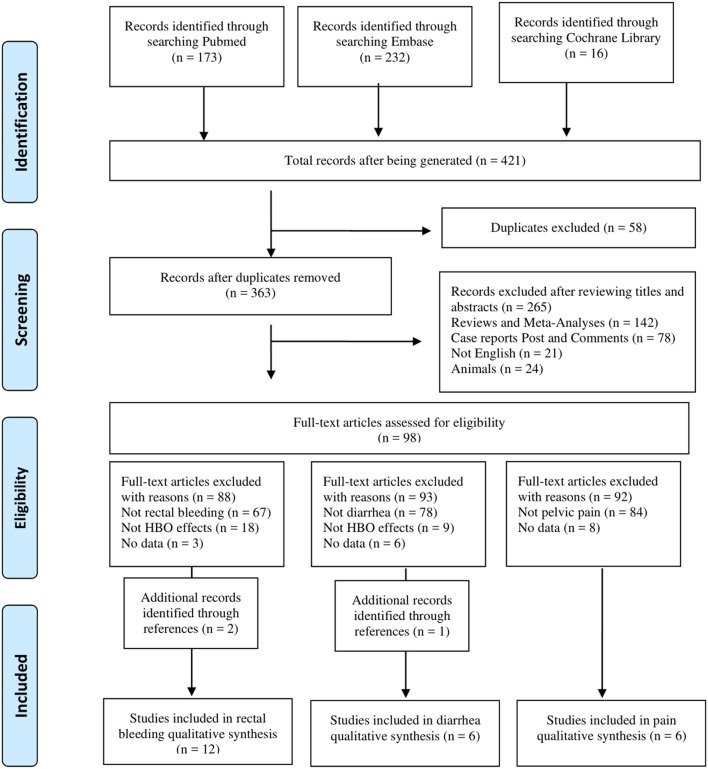
Flow chart of literature search.

The pelvic radiation-induced gastrointestinal complications include multiple symptoms. To evaluate the effects of HBO on different symptoms after radiation, two reviewers screened and classified 421 articles into different gastrointestinal symptom subgroups. Only three complications with sufficient articles were included for further analysis. Please refer to the PRISMA diagram (Figure 1) for the articles screening process. Data that met the criteria were extracted into a predefined standardized database in Microsoft Excel. The information collected included the following: the authors, year of publication, study design, study population, mean age, gender, time between radiotherapy and initial symptoms of radiation injury, hyperbaric oxygen (dose and number of sessions), outcome measures, and the follow-up time. Any disagreements were resolved through discussion.

The methodological quality of the included studies was evaluated by two authors independently according to the design of the study: the observational studies were appraised with the Newcastle-Ottowa Scale and the RCTs were assessed with Cochrane's Risk of Bias tool (27). Selection, comparability, exposure evaluation, or outcome evaluation of study population was assessed with the Newcastle-Ottowa Scale, while sequence generation, allocation concealment, blinding of participants and personnel, blinding of outcome assessors, incomplete outcome data, selective outcome reporting, other sources of bias, and overall risk of bias were evaluated with the Cochrane Collaborations tool.

### Statistical Analysis

R: A Language and Environment for Statistical Computing software was used to assess the normality of included data. Five methods of data transformation (“PRAW,” “PLN,” “PLOGIT,” “PAS,” and “PFT”) were applied to determine the effect of data transformations to the normality of data. It had been revealed that the data themselves are normally distributed; thus, no data transformation was applied for the subsequent analysis. STATA-12 (Version 12.0, StataCrop, College Station, TX, USA) was used for statistical analysis. The response rates (partial improvement and complete improvement) of different gastrointestinal complications to HBO treatment were estimated, forest plots were generated with a random-effects model, and confidence intervals (CI) were calculated as well. Since the occurrence rate ranges from 0 to 1, negative values were trimmed to 0, and values higher than 1 were set to 1. Heterogeneity was explored and quantified by the I^2^ statistic, and values ≥50% were interpreted as having substantial heterogeneity. Publication bias were inspected with funnel plots and further assessed by the Egger's regression asymmetry test.

## Results

### Literature Search

A total of 421 potential reports were filtered from databases, including PubMed, EMBASE, and Cochrane Library. After 58 articles were removed by the initial duplication check, titles and abstracts were reviewed and ineligible articles were removed according to the inclusion and exclusion criteria. Then eligible articles were classified by complications during the full-text review, and only the types of complications with the final included literature >5 were further analyzed: 12 studies of “rectal bleeding,” 6 studies of “diarrhea,” and 6 studies of “pain” were included in this meta-analysis. The details of the screening process are shown in Figure 1.

### Study Characteristics of Rectal Bleeding

After full text reviewing, 88 studies were removed and 2 additional articles were added. Finally, 12 studies were included in the “rectal bleeding” group for meta-analysis. A total of 330 participants were included in the selected studies, which were published from 1997 to 2016. The characteristics of the studies were displayed in Table 1: of the 12 studies selected for review, only 1 inclusion was a prospective study (36), two were RCTs (18, 28), and 9 selections were retrospective studies (29–35, 37, 38). The available data showed that the average age of the patients was 66 years old. There were four studies that only included male patients as participants, two studies that only included female patients, and the other six reports included both gender of patients. Only six studies reported the median time between pelvic radiation treatments and onset of initial symptoms. Among the studies included, at least 24 sessions of HBO treatments were performed per study, and the follow-up time is up to 39 months.

**Table 1:** Characteristics of included studies of rectal bleeding.

**References**	**Study design**	**Patient number**	**Age**	**Sex (m/f)**	**Time to symptom (months)**	**Resolution of bleeding with HBO**		**No improvement *n/N* (%)**	**Resolution of bleeding without HBO**		**No improvement *n/N* (%)**	**Dose of HBO (ATA)**	**Sessions of HBO**	**Follow-up after HBO (months)**
						**Complete recovery** ***n/N*** **(%)**	**Partial recovery** ***n/N*** **(%)**		**Complete recovery** ***n/N*** **(%)**	**Partial recovery** ***n/N*** **(%)**				
Clarke et al. (28)	RCT	119	–	Both (-)	–	5/63 (8%)	51/63 (81%)	7/63 (11%)	0/56 (0%)	35/56 (62.5%)	21/56 (37.5%)	2.0 vs. 1.1	30 and additional 10 treatments	25.1
Dall' Era et al. (29)	Retrospective	24	72	24/0	–	12/24 (50%)	7/24 (29%)	5/24 (21%)	–	–	–	2.4	36	13
Girnius et al. (30)	Retrospective	9	74	9/0	8.3	7/9 (78%)	2/9 (22%)	0/9 (0)	–	–	–	2.5	54	17
Glover et al. (18)	RCT	50	62	Both (-)	–	26/35 (74%)		9/35 (26%)	10/15 (67%)		5/15 (33%)	2.4 vs. 1.3	40	13.2
Jones et al. (31)	Retrospective	9	65	Both (-)	<24	4/9 (45%)	3/9 (33%)	2/9 (22%)	–	–	–	2.4	40	25
Marshall et al. (32)	Retrospective	53	65	Both (-)	–	24/53 (45%)	13/53 (25%)	16/53 (30%)	–	–	–	2.36	30 and additional 6–30 treatments	20
Mayer et al. (33)	Retrospective	9	71	9/0	7.8	3/9 (33%)	6/9 (67%)	0/9 (0)	–	–	–	2.2–2.4	30	11.1
Oliai et al. (34)	Retrospective	4	67	4/0	10.5	2/4 (50%)	1/4 (25%)	1/4 (25%)	–	–	–	2.0	38	39
Safra et al. (35)	Retrospective	6	64	0/6	10.1	5/6 (83%)	1/6 (17%)	0/6 (0)	–	–	–	2.0	27	-
Villegas et al. (36)	Prospective	19	52	0/19	–	16/19 (84%)		3/19 (16%)	Argon plasma coagulation			2.0–2.5	35 ± 5	3
Warren et al. (37)	Retrospective	11	65	Both (10/1)	8.4	6/11 (55%)	4/11 (36%)	1/11 (9%)	–	–	–	2.0 or 2.36	39	12
Woo et al. (38)	Retrospective	11	72	Both (17/1)	–	4/11 (36%)	1/11 (9%)	6/11 (55%)	–	–	–	2.0	24	14
Woo et al. (38)	Retrospective	4	72	–	–	0/4 (0%)	1/4 (25%)	3/4 (75%)	–	–	–	2.0	24	14
Woo et al. (38)	Retrospective	2	72	–	–	0/2 (0%)	1/2(50%)	1/2 (50%)	–	–	–	2.0	24	14

*RCT, randomized controlled trial; HBO, hyperbaric oxygen*.

### Overall and Subgroup Analysis

**Figure 2:**
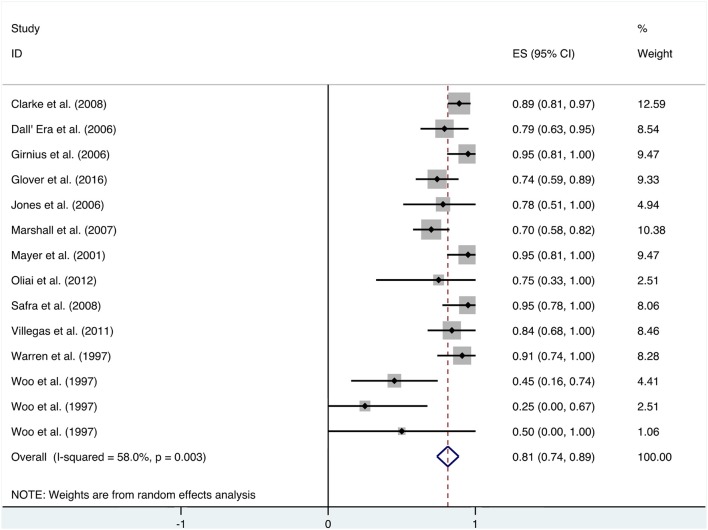
Forest plot of rectal bleeding.

**Figure 3:**
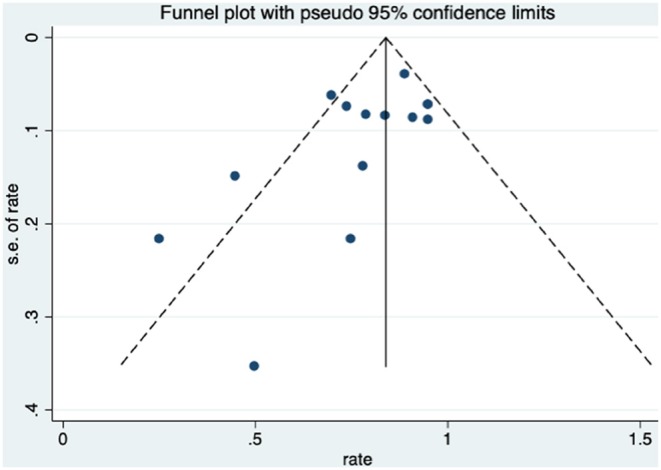
The funnel plot of the publication bias of rectal bleeding.

Patients received HBO treatments for rectal bleeding, and the rate of a partial or complete resolution was 0.81 (95% CI: 0.74–0.89) (Figure 2). The heterogeneity was analyzed with a random effect model (*I*^2^ = 58.0%, *P* = 0.003). The improvement rate of the control group without HBO treatment is 0.65 (95% CI: 0.55–0.74) (Supplementary Figure 1), and the heterogeneity was analyzed with a fixed effect model (*I*^2^ = 0%, *P* = 0.701). Publication bias was evaluated with Egger's test (*t* = −1.30, *P* = 0.218) and was considered insignificant. The funnel plot is shown in Figure 3.

**Figure 4:**
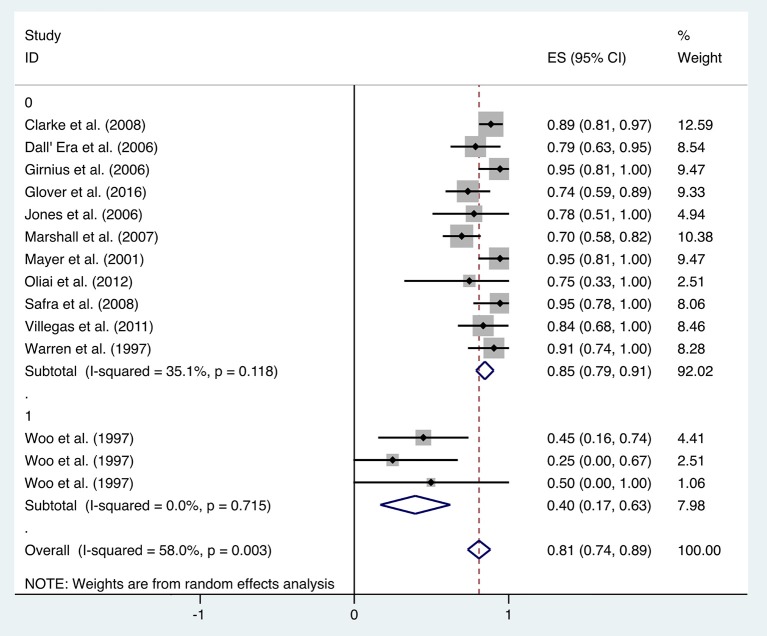
Forest plot of rectal bleeding based on evaluation criteria for symptom improvement.

Subgroup analysis was performed to investigate the factors that influenced heterogeneity. It was revealed that the improvement rate was influenced by the evaluation criteria for symptom improvement. Therefore, all included studies were divided into two subgroups: studies in the first subgroup define “improvement” by simply comparing the symptoms at the observation point and before treatment (transient improvement), while studies in the second subgroup define “improvement” as changes in symptoms lasting for at least 3 months (persistent improvement). Analysis on the 12 included studies focusing on transient improvement revealed a mean improvement rate of 0.85 (95% CI: 0.79–0.91, *I*^2^ = 35.1%), whereas the improvement rate in the studies focusing on persistent improvement was 0.40 (95% CI: 0.17–0.63, *I*^2^ = 0%) (Figure 4). Subgroup analyses were also performed by classifications including age, quantities of HBO sessions, and follow-up time, but no statistical significance or reduced heterogeneity was observed (Table 1).

### Study Characteristics of Diarrhea

Regarding diarrhea, 93 studies were removed after full text reviewing and one additional article was added. Finally, six studies were included for meta-analysis. There were a total of 188 participants included in the analysis, and the characteristics of the studies were demonstrated in Table 2: two inclusions were RCTs (18, 28) and the other four were retrospective studies (31, 32, 37, 38). There were four male patients and one female patient included in Warren et al., but in other studies, the gender distribution was unclear. Mean sessions of HBO treatments were listed in Table 2, which varied from 24 to 60. The reported follow-up time in Clarke et al. was an average time (25.1 months), but in all the other four studies, the median time (from 12 to 25 months) was reported (Table 2).

**Table 2:** Characteristics of included studies of diarrhea.

**References**	**Study design**	**Patient number**	**Age**	**Sex (m/f)**	**Time to symptom (months)**	**Resolution of diarrhea with HBO**		**No improvement *n/N* (%)**	**Resolution of diarrhea without HBO**		**No improvement *n/N* (%)**	**Dose of HBO (ATA)**	**Sessions of HBO**	**Follow-up after HBO (months)**
						**Complete recovery** ***n/N*** **(%)**	**Partial recovery** ***n/N*** **(%)**		**Complete recovery** ***n/N*** **(%)**	**Partial recovery** ***n/N*** **(%)**				
Clarke et al. (28)	RCT	119	–	Both (-)	–	5/63 (8%)	51/63 (81%)	7/63 (11%)	0/56 (0%)	35/56 (62.5%)	21/56 (37.5%)	2.0 vs. 1.1	30 and additional 10 treatments	25.1
Glover et al. (18)	RCT	39	62	Both (-)	–	14/23 (61%)		9/23(39%)	12/16 (75%)		4/16 (25%)	2.4 vs. 1.3	40	13.2
Jones et al. (31)	Retrospective	5	65		<24	1/5 (20%)	3/5 (60%)	1/5 (20%)	–	–	–	2.4	40	25
Marshall et al. (32)	Retrospective	12	65	Both (-)	–	4/12 (33%)	3/12 (25%)	5/12 (42%)	–	–	–	2.36	30 and additional 6–30 treatments	20
Warren et al. (37)	Retrospective	5	68	Both (4/1)	8.4	4/5 (80%)	1/5 (20%)	0/5 (0)	–	–	–	2.0 or 2.36	32	12
Woo et al. (38)	Retrospective	8	72	Both (-)	-	2/8 (25%)	2/8 (25%)	4/8 (50%)	–	–	–	2.0	24	14

*RCT, randomized controlled trial; HBO, hyperbaric oxygen*.

### Overall Analysis and Subgroup Analysis

**Figure 5:**
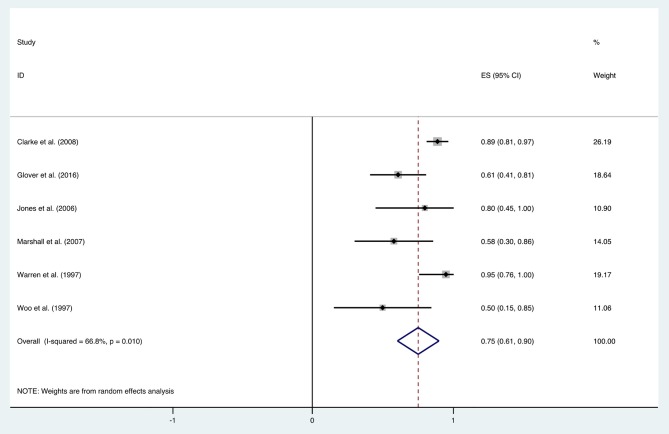
Forest plot of diarrhea.

**Figure 6:**
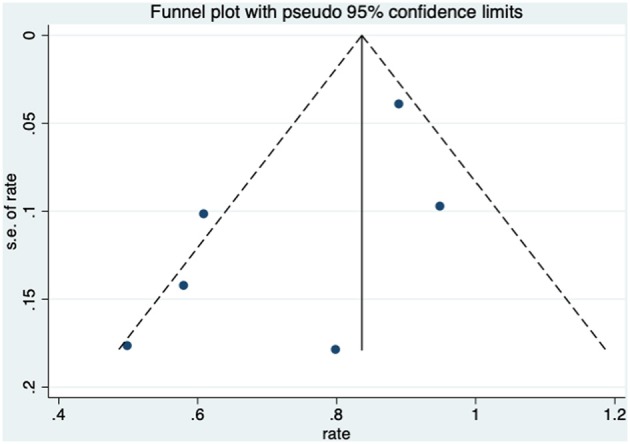
The funnel plot of the publication bias of diarrhea.

The improvement rate of patients who received HBO treatments for diarrhea was 0.75 (95% CI: 0.61–0.90) as shown in Figure 5. The heterogeneity was evaluated with a random effect model (*I*^2^ = 66.8%, *P* = 0.010). The improvement rate of the control group without HBO treatment is 0.65 (95% CI: 0.55–0.76) (Supplementary Figure 2), and the heterogeneity was analyzed with a fixed effect model (*I*^2^= 5.8%, *P* = 0.303). Publication bias was assessed with Egger's test (*t* = −1.77, *P* = 0.152), and there was no significant publication bias. The funnel plot was shown in Figure 6.

**Figure 7:**
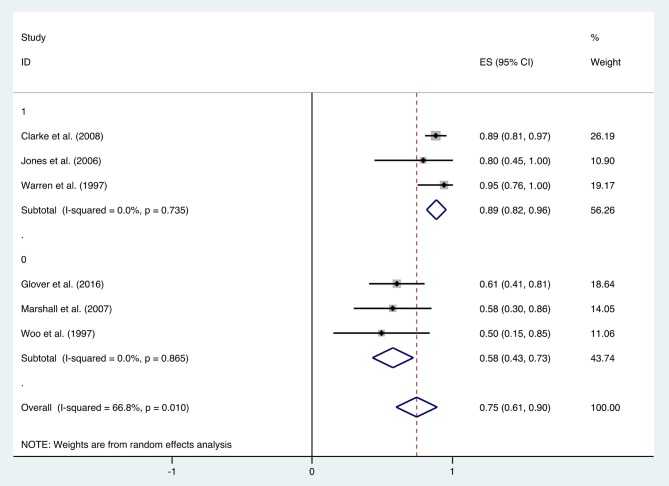
Forest plot of diarrhea based on follow-up time.

With the significant results of heterogeneity test, the factors influencing heterogeneity were further investigated. It was revealed that the improvement rate depended on the follow-up duration. Six included studies were divided into two subgroups: studies in the first subgroup had either short- or long-term follow-ups (≤ 12 months or ≥24 months), in which the mean improvement rate of HBO treatments was 0.89 (95% CI: 0.82–0.96), while studies in the second subgroup had follow-ups ranging from 12 to 24 months, which indicated a mean improvement rate of 0.58 (95% CI: 0.43–0.73) (Figure 7). Subgroup analysis for age and quantities of HBO sessions was also performed, which revealed no reduced heterogeneity (Table 2).

### Overall Analysis and Subgroup Analysis

**Figure 8:**
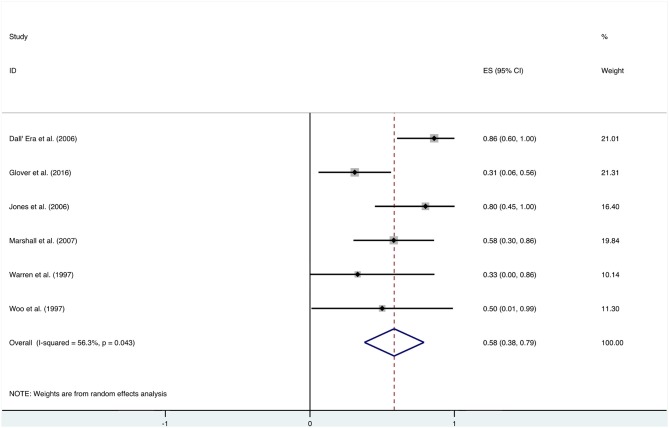
Forest plot of pain.

**Figure 9:**
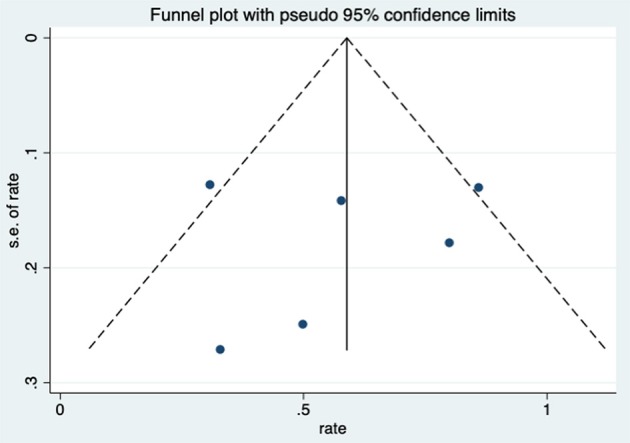
The funnel plot of the publication bias of pain.

After HBO treatments, the improvement rate of pain was 0.58 (95% CI: 0.38–0.79) (Figure 8). The heterogeneity was detected with a random effect model (*I*^2^ = 56.3%, *P* = 0.043). Publication bias was assessed with Egger's test (*t* = 0.09, *P* = 0.933), with no statistical significance detected. The funnel plot is shown in Figure 9.

**Figure 10:**
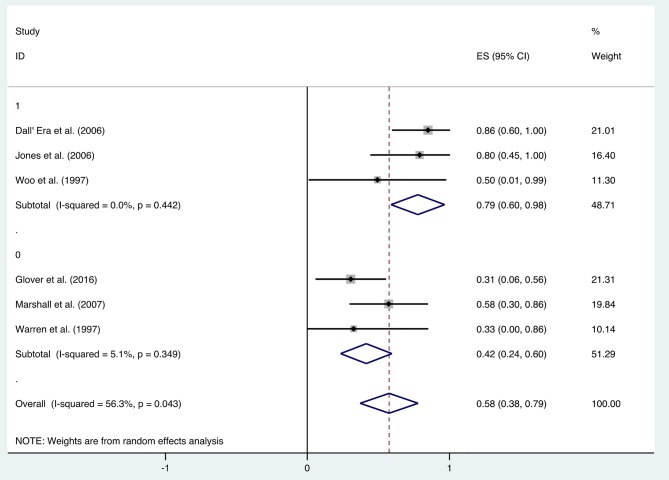
Forest plot of pain based on the scoring system.

Additional analysis identified the marking strategy as the factor that influenced heterogenicity. The included studies were divided into two subgroups: assessed only by clinical pain symptom relief or by a comprehensive scale that included pain evaluation. Three studies were assessed only by the relief of pain, resulting in the mean improvement rate of 0.79 (95% CI: 0.60–0.98) (Figure 10). Meanwhile, the other three studies assessed by comprehensive scale showed a mean improvement rate of 0.42 (95% CI: 0.24–0.60) (Figure 10). Other subgroup analyses classified by age, quantities of HBO sessions, or follow-up time were also performed, but no significant contribution was identified for these factors to explain heterogenicity in these studies (Table 3).

**Table 3:** Characteristics of included studies of pain.

**References**	**Study design**	**Patient number**	**Age**	**Sex (m/f)**	**Time to symptom (months)**	**Resolution of pain with HBO**		**No improvement *n/N* (%)**	**Resolution of pain without HBO**		**No improvement *n/N* (%)**	**Dose of HBO (ATA)**	**Sessions of HBO**	**Follow-up after HBO (months)**
						**Complete recovery** ***n/N*** **(%)**	**Partial recovery** ***n/N*** **(%)**		**Complete recovery** ***n/N*** **(%)**	**Partial recovery** ***n/N*** **(%)**				
Dall' Era et al. (29)	Retrospective	7	71	7/0	–	0/7 (0)	6/7 (86%)	1/7 (14%)	–	–	–	2.4	36	13
Glover et al. (18)	RCT	39	62	Both (-)	–	4/13 (31%)		9/13 (69%)	5/5 (100%)		0/5 (0)	2.4 vs. 1.3	40	13.2
Jones et al. (31)	Retrospective	5	65	–	<24	3/5 (60%)	1/5 (20%)	1/5 (20%)	–	–	–	2.4	40	25
Marshall et al. (32)	Retrospective	12	65	Both (-)	–	4/12 (33%)	3/12 (25%)	5/12 (42%)	–	–	–	2.36	30 and additional 6–30 treatments	20
Warren et al. (37)	Retrospective	3	70	Both (3/0)	8.4	0/3 (0)	1/3 (33%)	2/3 (67%)	–	–	–	2.0	20	20
Woo et al. (38)	Retrospective	4	72	Both (-)	–	1/4 (25%)	1/4 (25%)	2/4 (50%)	–	–	–	2.0	24	14

*RCT, randomized controlled trial; HBO, hyperbaric oxygen*.

## Discussion

### Summary of Results

In the current study, the effectiveness of HBO treatment on alleviating pelvic radiotherapy-induced gastrointestinal effects was investigated. The results revealed that HBO treatment might have the potential to alleviate rectal bleeding, diarrhea, and pain. Other symptoms related to radiation-induced gastrointestinal adverse effects were not further analyzed due to insufficient quantities of qualified studies. More studies are definitely needed in these aspects.

### Rectal Bleeding

When HBO treatment was applied for rectal bleeding alleviation, the overall improvement rate was 0.81 (95% CI: 0.74–0.89, *I*^2^ = 58.0%, Figure 2), which was higher than the baseline improvement rate from the control group without HBO treatment (Supplementary Figure 1), suggesting a therapeutic effect on rectal bleeding. While subgroup analyses revealed no significant contributions of age, quantities of HBO sessions, or follow-up time to the heterogeneity, the evaluation criteria for symptom improvement were indeed identified as the resource of heterogeneity in the analysis. Subgroup analysis taking the improvement criteria into account revealed substantial changes in the improvement rate (0.85 vs. 0.4) as indicated in Figure 4. A highly likely explanation for the dramatic differences in improvement rates is the more stringent standards for “improvement” in the studies of the subgroup, which takes “persistent improvement” (improve for at least 3 months) as the criterion for improvement, in comparison to the studies in the other subgroup, which takes “transient improvement” (improve at the moment of the study). This is a warning to the physicians that attention should be paid to not only the transient improvement of the symptoms but also the maintenance of improvement. The lower part of the funnel plot in Figure 3 is asymmetric, but the result of Egger's regression asymmetry test indicated that there were no publication biases.

Although this review focused on the improvement rates of symptoms in patients, studies involving control groups are still of great concern. In a randomized, double-blind, sham-controlled phase three trial, Inflammatory Bowel Disease Questionnaire (IBDQ) showed no significant differences between HBO and the control group on the proportion of patients with rectal bleeding improvement (74.3 vs. 66.7%, *P* = 0.58), and the authors concluded no evidence supporting benefits from hyperbaric oxygen therapy in patients with rectal bleeding (18). This report had aroused a considerable debate: Teguh et al. (25) addressed several drawbacks and pointed out the importance of early HBO intervention. Clarke et al. (28) attempted to reconcile this result, which contradicts to their findings, and also proposed that early interventions were critical to maximize the potential benefits (20). Wallington (39) and Bennett (19) reminded numerous sources of potential bias, including a high level of case selection and missing data during the study. As mentioned above, an earlier multinational RCT reported a significant increase in the improvement rate relative to the control group (89 vs. 62.5%, *P* = 0.0009) (28). More data from high-quality RCT studies based on larger populations are urgently needed for a more convincing conclusion. Based on our results, HBO might be effective in alleviating the symptoms investigated, but the duration of effect needs to be further observed and clarified. Additionally, a prospective study compared the effects of HBO and argon plasma coagulation (APC) on chronic radiation proctopathy-associated recurrent rectal bleeding. The authors reported that APC and HBO were both effective and safe, while the clinical response onset was faster in the APC group. However, two patients in the APC group had persistent rectal bleeding and were considered treatment failures, and then they were referred for HBO, and both had clinical improvement thereafter (36), suggesting the potential roles of HBO for refractory rectal bleeding treatment.

### Diarrhea

Recently, Lawrie et al. reviewed different interventions for radiotherapy-induced diarrhea alleviation, including pharmaceutical and non-pharmaceutical treatments, new methods (radiotherapy techniques), and other aspects of delivering radiotherapy. As only RCTs with a sample size of 20 or more were included in their review, only one study with HBO treatment qualified; thus, no analysis was performed. In the current study, more existing literatures were summarized for the assessment of the effectiveness of HBO against radiotherapy-induced diarrhea, which suggests that further investigation regarding the clinical application of HBO against radiation-induced diarrhea is necessary. Our data indicated that HBO might be beneficial to the patients suffering from diarrhea as the improvement rate was 0.75 (95% CI: 0.61–0.90), which was higher than the baseline improvement rate from the control group without HBO treatment (Supplementary Figure 2), suggesting a therapeutic effect on diarrhea. However, symptoms may reoccur in the duration of treatments, as the improvement rate remarkably decreased to 0.58 (95% CI: 0.43–0.73) when the follow-up time was set between 1 and 2 years. Overall, it is still generally beneficial in the long run, but physicians should inform patients prior to treatment that diarrhea may recur during 1–2 years after HBO treatments. Additionally, regular follow-up is important to those patients with diarrhea symptoms, and clinicians should pay attention to the possibility of recurrence after 1-year follow-up especially. Physicians could administer appropriate symptomatic treatment based on the severity of symptoms to help patients successfully overcome this period without further severe complications.

### Pain

In addition to the included articles, there was an RCT study from Shao et al. on pelvic pain that deserves attention. Although the report was not included in this review as the improvement rate was mainly calculated based on the alleviation rate of hematuria, they also evaluated the visual analog scale (VAS) of pelvic pain before and after the HBO treatments. The results showed a significant decrease in the VAS from baseline (2.5 ± 2.24) to 6 months (1.6 ± 1.79, *P* < 0.01), 12 months (1.6 ± 1.88, *P* < 0.01), and 18 months (1.35 ± 1.69, *P* < 0.01) after the HBO treatment (40). It indicated that HBO-induced alleviation on pelvic pain is persistent for at least 18 months. On the other hand, the selection of the evaluation system was revealed as the major source of heterogeneity, since the improvement rate was 0.79 (95% CI: 0.60–0.98) when only evaluated via clinical symptom vs. 0.42 (95% CI: 0.24–0.60) when scored by comprehensive scale.

To the best of our knowledge, the comprehensive scale such as the late effects of normal tissues: subjective, objective, management, analytic (LENT-SOMA) scale is more comprehensive and includes multiple endpoints evaluation. However, regarding the evaluation of pain management, it may not be the best choice, since factors other than pain (mucosa loss, stool frequency, bleeding, etc.) may affect the results. Moreover, the improvement of pain is not easy to be quantified in the comprehensive scale, which might lead to the underestimation of the treatment effects of HBO and patients' potential benefits from the treatments. In summary, the effectiveness of HBO treatment in pain management should not be ignored when comprehensive scale evaluation did not give promising results. In clinical work, the patient's quality of life is highly associated with pain management, and the potential of HBO treatment regarding this endpoint seems to be promising.

### Potential Biases and Limitations

Although efforts had been made to locate all available data by reviewing the references of qualified studies, possible publication bias might still present. Trials that failed to show any improvement in gastrointestinal symptoms after HBO treatment might have not been published, limiting the information availability. Moreover, the language restriction applied in this review could have increased the potential risk of publication bias as well.

There are some limitations to the current study. Among all the articles included in the three symptoms analyzed, there was only one prospective study and two RCTs, making it difficult to determine how much of reported improvement is attributable to HBO rather than the placebo effect. Although we presented the improvement rates in control group as a baseline data in supplementary materials, there could be some bias as only two RCTs were included in this work. And since the population of included studies was small, additional large-scale studies are needed to increase the quality of data. Furthermore, there are several methods of assessing the degree of symptoms, adding complexity in cross-study comparisons.

## Conclusion

HBO treatment might have the potential to improve radiotherapy-related gastrointestinal complications, including rectal bleeding, diarrhea, and pain, but more data are needed for further conclusions. Other symptoms were not further analyzed, as the number of studies was insufficient. More large-scale and prospective study is needed.

## Author Contributions

JY and JD contributed to the conception and design of the study. JY and RW assessed the studies and extracted the data. LS performed the statistical analysis. JY wrote the first draft of the manuscript. YL, QL, ML, and CZ wrote the sections of the manuscript. All authors contributed to manuscript revision, read, and approved the submitted version.

### Conflict of Interest

The authors declare that the research was conducted in the absence of any commercial or financial relationships that could be construed as a potential conflict of interest.

## Supplementary Material

Supplementary Figure 1 Forest plot of rectal bleeding without HBO treatments.

Supplementary Figure 2 Forest plot of diarrhea without HBO treatments.
