# Supplementary figures and images for: The Effects of Hyperbaric Oxygen Therapy on Pelvic Radiation Induced Gastrointestinal Complications (Rectal Bleeding, Diarrhea, and Pain): A Meta-Analysis
Source: Front Oncol. 2020 Apr 9;10:390. doi: 10.3389/fonc.2020.00390 (PMC7160697; doi:10.3389/fonc.2020.00390)

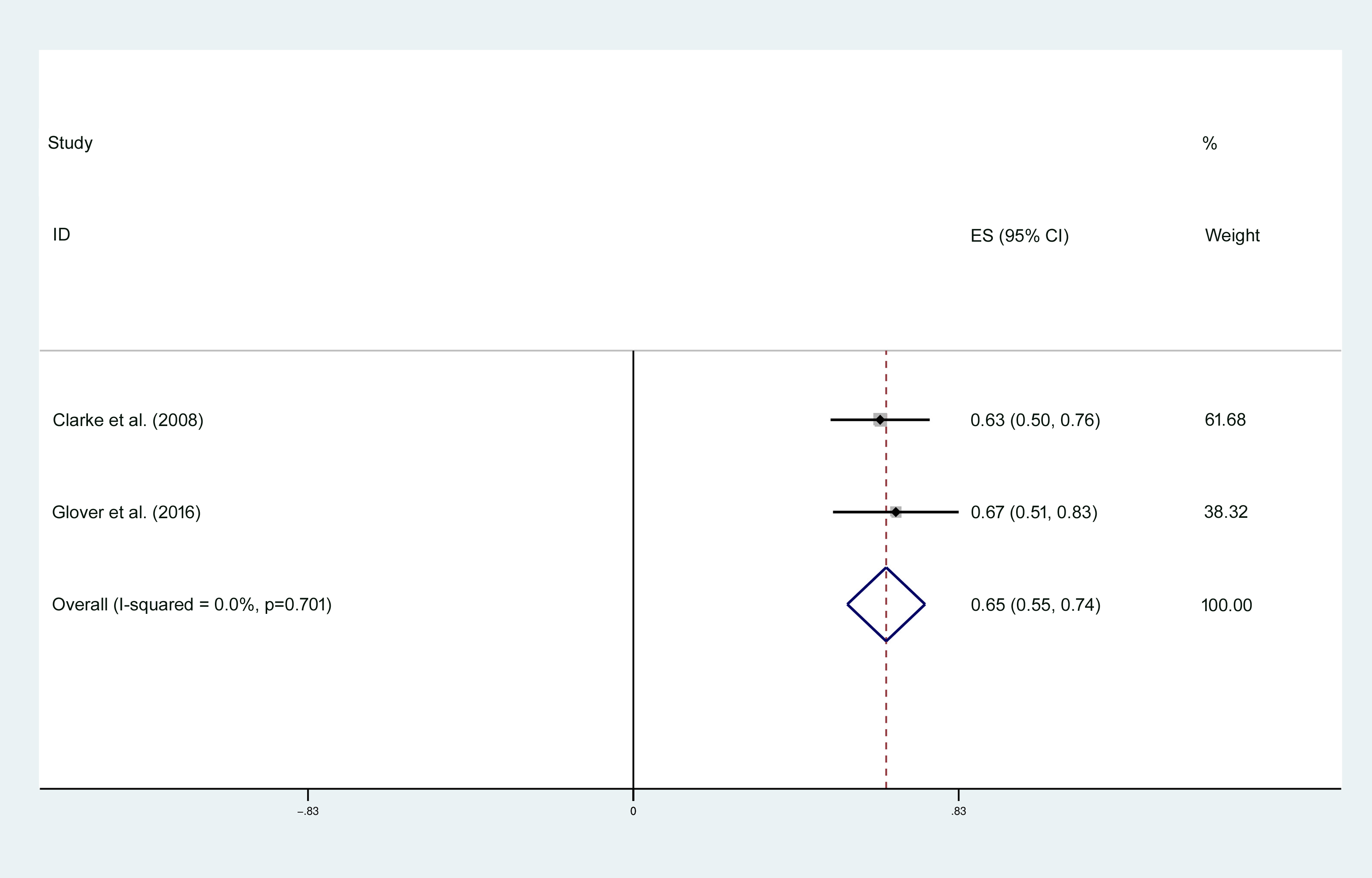

Supplement: Supplementary Figure 1 — Forest plot of rectal bleeding without HBO treatments. [file Image_1.TIF]

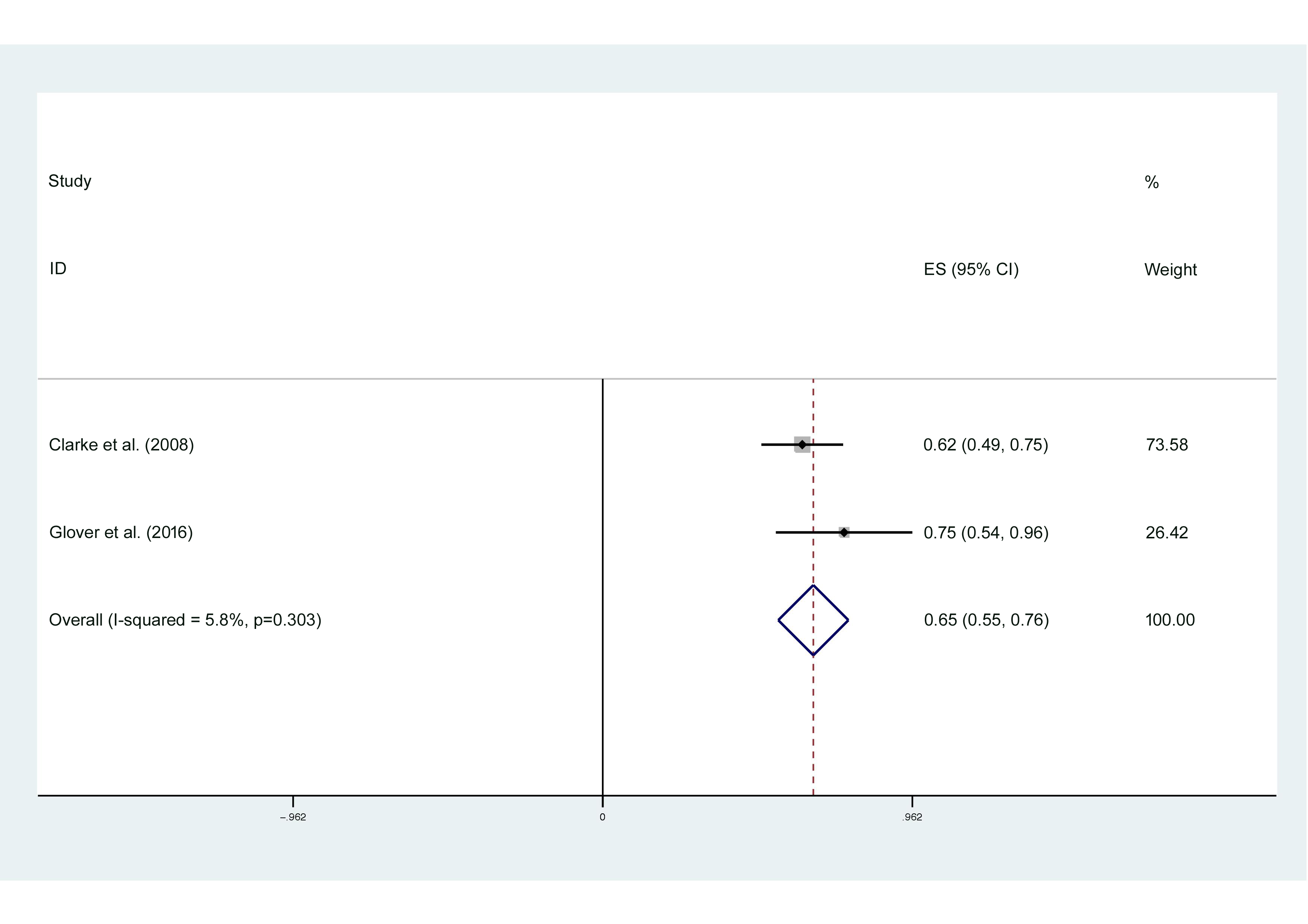

Supplement: Supplementary Figure 2 — Forest plot of diarrhea without HBO treatments. [file Image_2.TIF]
